# Exploring the Characteristics of Online Counseling Chat Services for Youth in Europe: Web Search Study

**DOI:** 10.2196/67949

**Published:** 2025-08-20

**Authors:** Irati Higuera-Lozano, Virvatuli Uusimäki, Tuuli Pitkänen, Elke Denayer, Alexis Dewaele, Katalin Felvinczi, Lien Goossens, Zsuzsa Kaló, Mónika Rényi, María Cabello

**Affiliations:** 1 Department of Psychiatry, Autonomous University of Madrid, Arzobispo morcillo, 4, Madrid, 28029, Spain, 34 914975988; 2 Fundación de Investigación Biomédica del Hospital Universitario La Princesa (IIS-Princesa), Madrid, Spain; 3 Finnish Youth Research Society, Helsinki, Finland; 4 Department of Clinical, Experimental, and Health Psychology, Faculty of Psychology and Educational Sciences, Ghent University, Ghent, Belgium; 5 Institute of Psychology, ELTE Eötvös Loránd University, Budapest, Hungary; 6 Instituto de Salud Carlos III., Centro de Investigación Biomédica en Red en Salud Mental (CIBERSAM), Madrid, Spain

**Keywords:** online chat, text-messaging, helpline, youth, mental health, Europe

## Abstract

**Background:**

Online counseling chat services are increasingly used by young people worldwide. A growing body of literature supports the use and effectiveness of these services for adolescent mental health. However, there is also a need to provide an overview of the main existing resources to identify unmet needs and gaps in the field.

**Objective:**

This study aims to provide an overview of existing online counseling chat services targeting individuals aged 12‐30 years in 4 European countries (Belgium, Finland, Hungary, and Spain), and to identify potential needs and gaps by comparing the collected data with recognized quality standard criteria that define the best practices in the field of counseling.

**Methods:**

A web search was conducted in the 4 participating countries using the same keywords to identify the main chat services. The final selection of chat services was made using a stratified purposive sampling method. A common data extraction database was developed to record information from these websites. Finally, the extracted information was compared against the fulfillment of 7 selected criteria from the Child Helpline International Quality Standards Framework. Additionally, certain chat characteristics were compared with the number of Child Helpline Quality Standard criteria fulfilled.

**Results:**

The search identified a total of 66 service providers offering 71 different chat services. Nongovernmental organizations accounted for more than half of the total service providers42 of 66 (64%). Additional helplines, such as hotlines, were also available through 54 of 66 (82%) service providers. Artificial intelligence tools were incorporated into 6 of 66 (9%) chat services. Differences were observed between countries; for example, the use of volunteers as counselors was predominant in Hungary and Belgium. Topic-specific chat services were common in Belgium and Spain, whereas in Finland and Hungary, chat services generally welcomed a wide range of topics for young people to discuss. Comparisons with Child Helpline International’s recommendations revealed some gaps—for example, only 9 of 71 (13%) chat services operated 24 hours a day, and only 10 of 71 (14%) offered interactions in minority groups or foreign languages. Additionally, the use of free social media platforms for chat services was prevalent in some countries, which could compromise users’ privacy. Being part of the Child Helpline International consortium was marginally associated with meeting a higher number of standard criteria (β coefficient 1.55; *P*=.08).

**Conclusions:**

This study provides a comprehensive overview of existing online chat counseling services in 4 European countries. Our findings suggest that some existing chat services for young people could be improved in areas such as accessibility, data security, and the inclusion of vulnerable groups.

## Introduction

The mental health of adolescents and young people is a global concern. Recent data indicate that 59% of Europeans aged 15‐24 years reported experiencing some form of emotional or psychological issue in the past year [1]. According to some reports, factors such as repeated economic crises, unemployment, the normalization of economic precarity, climate change, fears of conflict, and the COVID-19 pandemic have affected the mental health of young European people “somewhat” or “a lot” [2]. While most European countries provide universal health care coverage, formal entitlement does not always guarantee actual access to care. Various obstacles, such as long waiting times, bureaucratic procedures, and geographical barriers, can hinder access to mental health services [3]. Consequently, an increasing number of young individuals are turning to online platforms for support with their psychosocial challenges [4].

Compared with previous generations, online mental health support may be particularly appealing to youth [5]. Some studies suggest that young people may feel less judged in virtual environments [6], experience greater self-sufficiency [7], and enjoy increased control and autonomy [8], enabling them to seek help without disclosing their needs to parents, peers, or others [9].

Among the various digital support services available, real-time online counseling chat services (OCCSs) have unique characteristics that distinguish them from other forms of teletherapy or digital assistance. One notable feature is the “online disinhibition effect,” where users are more likely to disclose personal information due to reduced behavioral inhibitions in the online environment. This effect is driven by factors such as dissociative anonymity, invisibility, and authority minimization, among others [10]. Furthermore, users can engage in chat conversations from virtually anywhere and discuss sensitive topics without the need for a private setting. This flexibility allows them to control the pace and rhythm of the interaction [11,12]. In addition, research by Cohen et al [11] suggested that young people often prefer text-based communication over verbal exchanges (such as phone calls), which they may find intimidating. Existing literature on OCCSs has focused on analyzing the usefulness of these services in terms of effectiveness [13], predicting users’ satisfaction [14], identifying peak contact hours [15], profiling repeated users [16], and reporting relevant insights from the experiences of both counselors and users [17,18]. However, to our knowledge, no study has identified or explored the characteristics of existing OCCSs across different countries.

Child Helpline International is a global organization dedicated to strengthening child protection systems worldwide to improve the quality of responses to children and young people in need of protection, support, and guidance, and to advocate for their rights [19]. According to data published by Child Helpline International, online chat counseling services are currently the second most popular form of helpline among adolescents and children, after telephone calls [4]. The organization has also issued recommendations to ensure the quality of helplines for children and youth, including OCCSs [20].

Gathering relevant information about existing services and identifying potential gaps is a crucial first step in developing high-quality services that can meet the growing demand for mental health support [21].

Therefore, this study aimed to (1) identify primary service providers offering OCCSs that support the mental health and well-being of young people aged 12-30 in 4 European countries; (2) collect relevant information about these services using published data available on their websites; (3) identify potential unmet needs by comparing the available information with a selected set of quality criteria established by Child Helpline International; and (4) examine whether certain characteristics of service providers are associated with meeting a greater number of quality criteria. We hypothesize that organizations affiliated with the Child Helpline International network are more likely to meet a higher number of quality criteria than those that are not part of the consortium, and that more recently established chat services may also comply with a greater number of these criteria.

## Methods

### Procedure

This study is part of the Crisis Help and Assistance for Youth during Challenging Times (CH@T-YOUTH) project, launched under the ERASMUS+ framework as a Cooperation Partnership in youth and co-funded by the European Union (from September 1, 2023, to January 31, 2026). The primary aim of the project is to generate knowledge about OCCSs that support youth mental well-being. In addition, the project seeks to facilitate the exchange of knowledge among service providers and youth workers, and to promote ethical reflection aimed at enhancing the quality of both emerging and existing nonprofit OCCSs across Europe.

The initial phase of the study aimed to deepen the understanding of OCCSs for youth across 4 participating countries, namely, Belgium, Finland, Hungary, and Spain, representing diverse geographical regions within Europe. The first step, as referred to in this article, was to identify existing services and extract data on their characteristics, focusing only on publicly available information from websites, to assess the level of information accessible to users. To achieve this, a common, step-by-step methodology was applied. This methodology, used in previous studies [22], guided the compilation of a comprehensive country-wise catalog of the main chat service providers in the 4 participating countries.

### Service Inclusion and Exclusion Criteria

The inclusion criteria focused on organizations providing OCCSs to individuals aged 12-30, or to organizations where the majority of clients fell within this age range. Eligible services were required to target either the general youth population or specific vulnerable groups (such as Ukrainian refugees, minorities, or the LGBTIQ+ [Lesbian, Gay, Bisexual, Transgender, Intersex, Queer/Questioning, and others] community), with mental health and well-being as a primary or secondary outcome.

Online chat services that were not free of charge, or that functioned solely as referral services—providing users with relevant contact points without offering ongoing support—were excluded from this study. Organizations that solely offered other forms of text-based helplines, such as asynchronous messaging, email contact points, or chat services without real-time human interaction (ie, limited to artificial intelligence) were also excluded. However, organizations that provided additional services or helplines were included if their chat services met the specified inclusion criteria.

### Search Strategy

**Figure 1.**
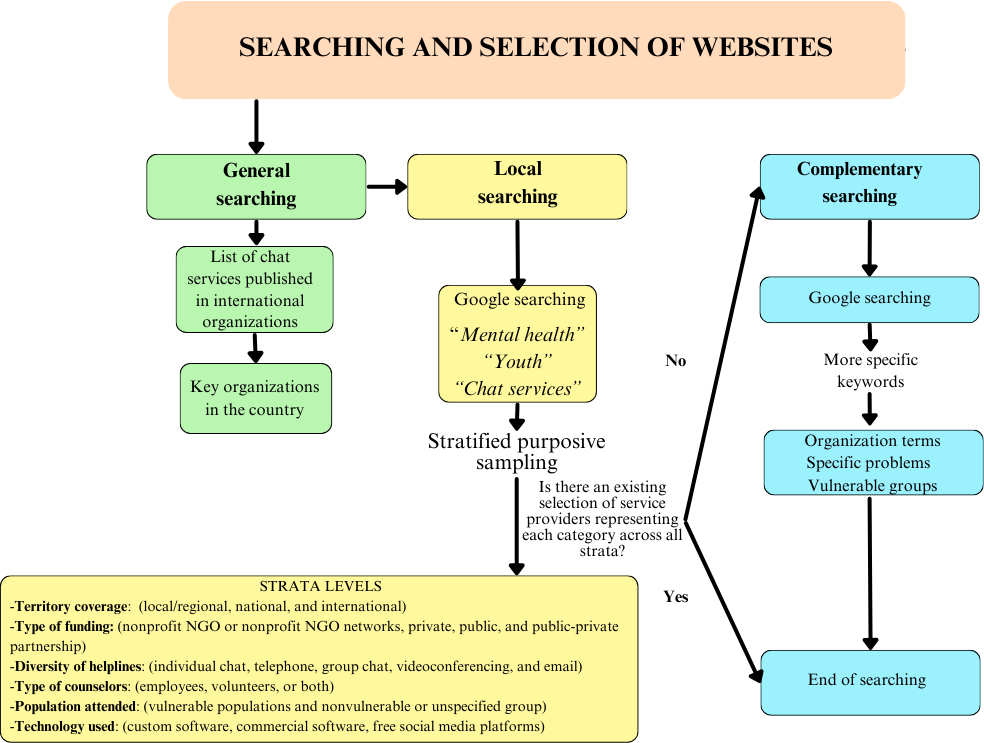
The process of searching and selecting websites. NGO: nongovernmental organization.

A web search study was conducted from March to May 2024. The process of searching and selecting websites is summarized in Figure 1. First, the websites of Child Helpline International, the International Association for the Prevention of Suicide, HelpGuide, Mental Health Europe, the Red Cross, and SOS Children’s Villages were consulted to identify key organizations in the target countries. In addition, an internet search was conducted using the Google web browser. Common keywords such as “mental health,” “chat online,” and “youth” were translated into local languages—Dutch, Finnish, Swedish, Hungarian, Spanish, Catalan, Basque, and Galician—to identify OCCS providers. In the case of Finland, keywords were also used in English. The internet search was not constrained by Google’s algorithms, as our goal was to replicate the type of search a typical user might perform when seeking help. The selection of service providers was guided by a stratified purposive sampling strategy, aimed at capturing major variations among the identified services, rather than identifying a common core, although common themes may still emerge during analysis [23]. The different strata levels that guided the selection of chat providers are shown in Figure 1. The mapping process was considered complete once all categories within the strata were filled, if possible, at least once.

If any of the levels was not covered, a complementary internet search was conducted using addition terms related to service providers (eg, foundation, association, organization), minority populations (eg, LGBTIQ+, refugee, immigrant) and key mental health issues (eg, anxiety, depression, eating disorders, loneliness, bullying; Figure 1).

### Data Extraction

To facilitate data collection from the websites, a common online database using Webropol (Webropol Oy) was developed and completed by researchers from each participating country (IHL, ED, MR, and VU). The database included information on the following variables: year the organization was founded (*before the 1990s, 1990‐2000, 2001‐2010, 2011‐2024);* type of funding (*nonprofit nongovernmental organization [NGO] or nonprofit NGO networks, private, public, and public-private partnership*); diversity of helplines offered (*individual chat, telephone, group chat, videoconferencing, and email*); type of counselors used (*employees, volunteers, or both*), number of counselors (*<50, 50‐250, and >250*); number of users served annually, based on the latest report; and availability of annual reports detailing the organization’s activities (*yes, or no information/not available*). In addition, the websites of all service providers were thoroughly explored for details about their chat services. The data collection captured the following information: the year the chat service was established (*before 2010; 2011‐2018, pre–COVID-19; 2019‐2021, COVID-19 period; and 2022‐2024, post–COVID-19 period*); coverage of the chat (*local/regional, national, or international*); age groups covered in the chat (*<18, >18, specific age range including minors and adults, everyone, or no information*); languages used in the chat (*official languages recognized by the government, minority or foreign languages, both official and minority/foreign languages, or English-only chat services*); technology employed in the chat (*custom software, commercial software, free social media platforms, or others*); chat availability (*24/7, specific hours 7 days a week, specific hours and days, other, or no information*); target populations (*youth with addiction issues, LGBTIQ+ community, individuals who experienced abuse or neglect, pregnant women, individuals who experienced gender or domestic violence, other vulnerable groups, or nonvulnerable/unspecified group)*; and specific issues addressed (*all topics, specific mental health problems, violence, addiction, other specific psychosocial issues, sexuality gender, 2 of the specific topics, or not reported*). In cases where websites provided incomplete information, no further contact was established, to reflect the level of information publicly available to users.

### Comparison With the Child Helpline International’s Core Quality Standard Framework

The data collected were compared with the Child Helpline International’s Core Quality Standards (CQS) framework [20]. The CQS framework was developed as a valuable reflection tool and reference point for quality management in child helplines. According to this framework, high-quality child helplines should be rights-based; advocate for children; facilitate meaningful participation of children and young people; be reliable; ensure safety and not cause harm; be accessible; child-centered; accountable; responsible; and effective in emergencies. The comparison focused on the following 3 CQS criteria: (1) *Advocating for children* (ie, the child helpline amplifies the voice of children by collecting, collating, and sharing data about children’s contacts); (2) *Safety—do not harm* (the child helpline should safely collect, record, and protect children’s information using a secure platform); and (3) *Accessibility* (the child helpline should be accessible, operate nationally and 24/7, and provide a safe, inclusive service to recognized marginalized groups such as minority language speakers, LGBTIQ+, refugees, or those in other vulnerable situations). Marginalized groups were defined as those facing additional barriers in accessing mental health services, including people using minority or foreign languages in the country. These groups were categorized based on the collected data and existing literature on vulnerable populations [20,24-26]. The fulfillment of the remaining CQS criteria could not be assessed using the publicly available data published on the websites. Finally, a total composite score ranging from 0 to 7 was created, reflecting the number of CQS criteria met by each service provider.

### Analyses

The characteristics of OCCS and their providers were described using frequencies and percentages, both overall and by country. The main findings were descriptively compared with the selected criteria from the CQS framework. This comparison allowed for the identification of potential gaps and provided an opportunity to evaluate the ecological validity of specific criteria. Finally, to assess whether certain characteristics of chat services were associated with meeting a higher number of the selected Child Helpline International quality criteria, a linear regression analysis was conducted. The analysis included the year the chat service was established and whether the service belonged to the Child Helpline International consortium (Yes/No) as independent variables.

## Results

### Characteristics of Service Providers

The main characteristics of the 66 service providers were examined (Table 1).

**Table 1.** Main characteristics of service providers extracted from their websites by total and country.

Variables	Total (n=66), n (%)	Belgium (n=23), n (%)	Finland (n=20), n (%)	Hungary (n=9), n (%)	Spain (n=14), n (%)
Year of foundation					
Before 1990s	32 (48)	12 (52)	11 (55)	1 (11)	8 (57)
1990‐2000	11 (17)	4 (17)	0 (0)	6 (67)	1 (7)
2001‐2010	5 (8)	0 (0)	1 (5)	1 (11)	3 (21)
2011‐2024	13 (20)	6 (26)	5 (25)	1 (11)	1 (7)
Not reported	5 (8)	1 (4)	3 (15)	0 (0)	1 (7)
Type of service provider					
Public	15 (23)	7 (30)	4 (20)	0 (0)	4 (29)
Private-public collaboration	6 (9)	1 (4)	0 (0)	0 (0)	5 (36)
Private	1 (2)	0 (0)	0 (0)	0 (0)	1 (7)
Nonprofit nongovernmental organizations or collaborations	42 (64)	15 (65)	15 (75)	8 (89)	4 (29)
Other	2 (3)	0 (0)	1 (5)	1 (11)	0 (0)
Type of helplines delivered					
Only individual chat	12 (18)	8 (35)	3 (15)	0 (0)	1 (7)
Telephone + chat	7 (11)	1 (4)	3 (15)	1 (11)	2 (14)
Group chat + chat	7 (11)	0 (0)	6 (30)	1 (11)	0 (0)
Telephone, email + chat	23 (35)	13 (57)	0 (0)	5 (56)	5 (36)
Telephone, email, videoconference + chat	7 (11)	1 (4)	1 (5)	1 (11)	4 (29)
Other + chat	10 (15)	0 (0)	7 (35)	1 (11)	2 (14)
Type of counselors					
Employees	21 (32)	6 (26)	10 (50)	1 (11)	4 (29)
Volunteers	16 (24)	9 (39)	1 (5)	5 (56)	1 (7)
Both	14 (21)	1 (4)	6 (30)	2 (22)	5 (36)
Not reported	15 (23)	7 (30)	3 (15)	1 (11)	4 (29)
Number of counselors					
<50	10 (15)	3 (13)	2 (10)	1 (11)	4 (29)
>50‐250	6 (9)	3 (13)	2 (10)	1 (11)	0 (0)
>250	2 (3)	2 (9)	0 (0)	0 (0)	0 (0)
Not reported/not available	48 (73)	15 (65)	16 (80)	7 (78)	10 (71)

The number of service providers ranged from 23 in Belgium to 9 in Hungary. Half of the providers identified were established before the 1990s. Nonprofit NGOs were the predominant type of service provider in most countries. Most organizations (54/66, 82%) also offered additional helplines beyond online chat services, with telephone support being the most common. The combination of telephone, email, and individual chat was particularly popular (accounting for 23/66, 35%, services). In addition, 6 out of 66 (9%) providers incorporated some form of artificial intelligence into their chat services. In Finland and Spain, counselors were typically a mix of both employees and volunteers, whereas services with mostly volunteers were characteristic in Hungary and Belgium. Of the 66 service providers, 48 (73%) did not report the number of counselors available in their organization.

### Characteristics of Chat Services

Table 2 shows the main characteristics of online chat counseling services.

**Table 2.** Main characteristics of chat services extracted from their websites by total and country.

Variables	Total (n=71), n (%)	Belgium (n=23), n (%)	Finland (n=21), n (%)	Hungary (n=9), n (%)	Spain (n=18), n (%)
Year of creation of the chat					
Before 2010	5 (7)	5 (22)	0 (0)	0 (0)	0 (0)
2011‐2018 (pre–COVID-19 period)	14 (20)	2 (9)	8 (38)	2 (22)	2 (11)
2019‐2021 (COVID-19 period)	10 (14)	2 (9)	1 (5)	4 (44)	3 (17)
2022‐2024 (post–COVID-19 period)	15 (21)	1 (4)	6 (29)	0 (0)	8 (44)
No information	27 (38)	13 (57)	6 (29)	3 (33)	5 (28)
Age of chat users					
<18 years	10 (14)	3 (13)	4 (19)	1 (11)	2 (11)
>18 years	6 (8)	2 (9)	1 (5)	1 (11)	2 (11)
Specific age range or period (including minors and adults)	26 (37)	2 (9)	14 (67)	2 (22)	8 (44)
Everyone	22 (31)	13 (57)	2 (10)	1 (11)	6 (33)
Not reported	7 (10)	3 (13)	0 (0)	4 (44)	0 (0)
Specific problems targeted					
All topics	32 (45)	5 (22)	16 (76)	6 (67)	5 (28)
Violence	10 (14)	3 (13)	2 (10)	1 (11)	4 (22)
Addiction	6 (8)	1 (4)	1 (5)	0 (0)	4 (22)
Sexuality, gender	4 (6)	3 (13)	1 (5)	0 (0)	0 (0)
Other specific mental health issues	5 (7)	3 (13)	0 (0)	0 (0)	2 (11)
Other specific psychosocial problems	10 (14)	7 (30)	1 (5)	0 (0)	2 (11)
Two of the specific topics	2 (3)	0 (0)	0 (0)	1 (11)	1 (6)
Not reported	2 (3)	1 (4)	0 (0)	1 (11)	0 (0)
Average users who attended the chat (last reported year)					
Median (range)	1181 (19‐33,151)	2356 (38‐8281)	2332 (91‐33,151)	194	899 (19‐3500)
Not reported	49 (69)	14 (61)	14 (67)	8 (89)	13 (72)

Some service providers offered multiple chat helplines, which explains the discrepancy between the number of service providers and the total number of chat helplines. While 27 of 71 (38%) organizations did not report when their OCCSs began, available data indicate an increase in the number of chat services over the past 2 years, particularly in Finland and Spain (Table 2). Some chat services (26/71, 37%) targeted both minors and adults within a defined age range, followed by services open to everyone (22/71, 31%) and those aimed specifically at minors (10/71, 14%) or adults (6/71, 8%); 32 of the 71 (45%) OCCSs were open to any topic or issue related to well-being, while the remaining services focused on specific areas such as violence or addiction. The number of users served in the last reported year ranged from 19 to 33,151.

### Comparison With the Child Helpline International’s Child Helpline International’s CQS Framework

The comparison of the collected data with the Child Helpline International’s CQS framework is presented in Table 3.

The results in Table 3 show that 40 of 71 (56%) service providers reported publishing data on their activity annually. Regarding the sharing of data on children’s contacts, only 22 of 71 (31%) OCCSs publicly reported the number of users.

In terms of accessibility, only 9 of 71 (13%) chat services were available 24/7, and almost half of the OCCSs were available less than 7 days a week and only during certain hours (Table 3). Regarding coverage, 30 of 71 (42%) OCCSs had national coverage, while 36 of 71 (51%) operated at the local or regional level. In terms of accessibility for marginalized or minority/vulnerable groups, 28 of 71 (39%) chat services specifically targeted these populations, primarily youth with addiction issues, followed by the LGBTIQ+ community, individuals who experienced abuse and neglect, pregnant women, and individuals who experienced gender-based violence (Table 3). Results also showed that only 10 of 71 (14%) OCCSs provided services in minority or foreign languages.

Another important point is that helplines should be safe. For example, it is not recommended to use platforms that do not protect users’ data. In general, the most commonly used technology in OCCSs was commercial and custom software developed by external companies. However, in countries such as Spain, most OCCSs (14/18, 78%) used social media.

The results show the relationship between the number of Child Helpline Quality Standard criteria met and certain chat service characteristics. Being part of the Child Helpline consortium was marginally associated with meeting a higher number of standard criteria (β coefficient=1.55; *P*=.08). However, the year the chat service was established was not significantly related to the number of criteria met (β coefficient=3.66; *P*=.94).

**Table 3.** Comparison between information available on websites and Child Helpline International’s Core Quality Standards framework.

Detailed standard	Total (n=71), n (%)	Belgium (n=23), n (%)	Finland (n=21), n (%)	Hungary (n=9), n (%)	Spain (n=18), n (%)
Advocating for children					
Report on helpline activities					
Yes, available	40 (56)	17 (74)	10 (48)	3 (33)	10 (56)
Not available (no information)	31 (44)	6 (26)	11 (52)	6 (67)	8 (44)
Number of users served in the most recent reporting year					
Yes	22 (31)	9 (39)	7 (33)	1 (11)	5 (28)
No	49 (69)	14 (61)	14 (67)	8 (89)	13 (72)
Accessibility					
Time availability					
24/7	9 (13)	0 (0)	0 (0)	0 (0)	9 (50)
Specific hours/7 days	11 (15)	2 (9)	3 (14)	3 (33)	3 (17)
Specific hours and days	35 (49)	15 (65)	9 (43)	6 (67)	5 (28)
Other	8 (11)	3 (13)	5 (24)	0 (0)	0 (0)
No information	8 (11)	3 (13)	4 (19)	0 (0)	1 (6)
Territory coverage^a^					
Local/regional	36 (51)	19 (83)	4 (19)	1 (11)	12 (67)
National	30 (42)	1 (4)	15 (71)	8 (89)	6 (33)
International	3 (4)	1 (4)	2 (10)	0 (0)	0 (0)
Not reported	2 (3)	2 (9)	0 (0)	0 (0)	0 (0)
Language of chat					
Official language	52 (73)	20 (87)	15 (71)	8 (89)	9 (50)
Minority/foreign language	1 (1)	0 (0)	1 (5)	0 (0)	0 (0)
Both official and minority/foreign languages	9 (13)	1 (4)	4 (19)	1 (11)	3 (17)
Other	1 (1)	0 (0)	1 (5)	0 (0)	0 (0)
No information	8 (11)	2 (9)	0 (0)	0 (0)	6 (33)
Vulnerable population					
Youth with addiction issues	7 (10)	1 (4)	1 (5)	1 (11)	4 (22)
LGBTIQ+^b^ collective	5 (7)	2 (9)	1 (5)	1 (11)	1 (6)
Individuals who experienced abuse or neglect	6 (8)	3 (13)	1 (5)	0 (0)	2 (11)
Pregnant women	4 (6)	3 (13)	0 (0)	0 (0)	1 (6)
Individuals who experienced gender and domestic violence	3 (4)	0 (0)	0 (0)	1 (11)	2 (11)
Other vulnerable group (including refugees)	3 (4)	1 (4)	2 (10)	0 (0)	0 (0)
Not vulnerable or unspecified group	43 (61)	13 (57)	16 (76)	6 (67)	8 (44)
Safety—do not harm					
Type of software used					
Custom software	16 (23)	3 (13)	0 (0)	2 (22)	11 (61)
Commercial software	22 (31)	16 (70)	3 (14)	3 (33)	0 (0)
Free social media	16 (23)	0 (0)	0 (0)	2 (22)	14 (78)
Other technology	3 (4)	1 (4)	1 (5)	0 (0)	1 (6)
Not reported	22 (31)	3 (13)	17 (81)	2 (22)	0 (0)

a Local/regional coverage means the service is mainly intended for users in a specific city, municipality, or region. National coverage refers to services accessible across the entire country. International coverage includes services that are accessible to users beyond one national context, sometimes across multiple countries.

b LGBTIQ+: Lesbian, Gay, Bisexual, Transgender, Intersex, Queer/Questioning, and others.

## Discussion

### Principal Findings

This study aimed to identify and collect relevant information about the main OCCSs in Belgium, Finland, Hungary, and Spain. A total of 66 service providers, comprising 71 different OCCSs, were identified and evaluated against Child Helpline International’s quality standards. Most services were NGOs with extensive helpline experience before the implementation of OCCSs. Key areas for further improvement were time accessibility, data security, and the coverage of certain vulnerable groups. Service providers that were part of the Child Helpline International consortium were marginally more likely to meet a higher number of quality standard criteria.

Our findings show that the number of chat services increased during and after the COVID-19 pandemic. This suggests that helpline providers have diversified their outreach methods, with chat services becoming an integral part of helpline offerings in the 4 countries studied. However, hotlines remained the most commonly provided form of support. This finding aligns with data reported by Child Helpline International [4].

Regarding the types of assistants attending the chat, our results showed that services were primarily staffed by paid personnel, followed by volunteers, and then a combination of both. Some studies have argued that lay volunteers may better relate to the experiences of callers [27]. However, the quality of support provided by volunteers and paid counselors has been found to be comparable [27]. By contrast, the lack of financial compensation for volunteers may contribute to higher intentions to leave, particularly in response to stressful situations [28].

Our study also identified potential gaps by comparing the publicly available information on web sources with the CQS framework provided by Child Helpline International [20]. While the framework advocates for 24/7 availability, it acknowledges that, when resources are limited, queries should at least be answered during consistent and reliable operating hours. Our findings showed that the majority of the existing OCCSs were available for fewer than 7 days a week and operated only during specific hours. Most organizations were NGOs; hiring staff is expensive, and finding volunteers to ensure 24/7 coverage is a challenge. In addition, previous studies have suggested that one-third of user contacts occur after 8 PM [29]. Therefore, if a 24/7 service is not feasible, identifying and covering peak hours based on young people’s schedules may be a more realistic approach in the context of limited resources. Our study also found that the few OCCSs operating 24/7 were mostly government-funded or operated through public-private partnerships, and typically staffed by paid professionals or a combination of staff and volunteers. Literature has shown that night shifts are among the tasks most strongly associated with elevated distress levels and the intention to resign among helpline volunteers [28]. Although concrete evidence is lacking, it is reasonable to assume that night shifts in 24/7 services are primarily covered by paid personnel. In resource-limited settings, an alternative approach to optimizing resource allocation and availability can be seen in Finland, where different service providers have collaborated to analyze peak usage times and adjust their operating hours accordingly [30].

Another critical point is that existing OCCSs should improve accessibility for minorities and vulnerable groups by studying and addressing help-seeking barriers specific to these populations [20]. One example is that some service providers offered dedicated chat services, apart from the general ones, tailored to the needs of these groups. Regarding specific vulnerable populations, the LGBTIQ+ community was represented in all countries, with at least one chat service aimed at them. This is a positive finding, as the LGBTIQ+ collective often perceives general-population helplines as inappropriate or unsupportive and typically prefers specialized services where they feel better understood [31]. Individuals with substance use issues were also broadly covered. Youth in particular often face stigma and shame when seeking help for substance use problems [32]. Specialized anonymous service tailored to their needs may therefore facilitate better access to care. However, other marginalized groups, such as migrant and refugee populations, were underserved, with only 1 chat service specifically tailored to them. This represents an important gap, as chat services could be particularly beneficial for these populations, who often face significant barriers to accessing traditional face-to-face health care services [6]. For example, concerns about deportation may prevent individuals, especially those with irregular status, from seeking assistance [33].

In addition, according to Child Helpline International, helpline services should be accessible to users who speak minority languages within the country. Our data indicated that only 10 of 71 (14%) OCCSs offered services in minority or foreign languages. Cooperation between European countries could enhance the coverage of OCCSs for vulnerable populations, such as migrants, refugees, and minorities. Some initiatives have already been established to support Ukrainian refugees, illustrating the potential of collaborative efforts to improve accessibility [34,35]. To conclude our discussion on accessibility, one rationale for service providers operating multiple chat helplines may be to enhance access to counseling services for marginalized groups. This approach aligns with Child Helpline International’s standards, which advocate tailoring OCCSs to meet the diverse needs of different populations.

Regarding the visibility of OCCSs, Child Helpline International recommends a national-level service [20]. However, in our study, half of the service providers had local or regional coverage, while the remainder were supported by national and international institutions. In the context of Europe—with its linguistic, cultural, and administrative diversity—local and regional services may be particularly well suited to respond to the specific needs of their target populations. For example, a local service may be more attuned to the social or cultural features of a specific area. Working together to establish networks among local and regional services could further improve visibility and help address the specific needs of users [30].

The Child Helpline International guidelines also emphasize the importance of ensuring privacy and security in OCCS conversations [20]. Low adherence to ethical standards regarding online privacy in counseling was previously reported by Chester and Glass [36], who found that 42% of online counselors did not use any encryption to protect user confidentiality. Our study showed that in some countries, such as Spain, free social media software was one of the platforms most commonly used by chat service providers. While such platforms may enhance accessibility for users, they can also compromise privacy. To ensure security in an ever-changing digital environment, it is essential to continuously evaluate ethical, technical, and usability considerations—and to learn from the practices of other OCCSs.

Another area for improvement is the systematic collection and publication of activity data, which can enhance visibility, inform policy design, and support funding allocation. Child Helpline International has specifically advocated for clear criteria in the analysis and reporting of contact trends [20]. Although more than half of the service providers published data regularly, most did not report information on the number of cases handled. This may stem from the absence of a long-term strategy and a reluctance to have their performance evaluated by third parties [37], particularly among smaller NGOs, where funding is often uncertain. Improving systematic data collection and ensuring transparency about the characteristics of OCCSs could significantly enhance visibility and help attract additional resources, including funding. This is particularly important because, while some countries have government-funded organizations, most service providers are NGOs that rely primarily on external donations.

Regarding the factors associated with meeting a greater number of Child Helpline International’s recommendations, the year the chat service was created was not significantly related. Only membership in Child Helpline International showed a marginal association with meeting a higher number of criteria. It is possible that having sufficient funding and resources is more closely linked to meeting the standards set by Child Helpline International. Smaller, local, and regional service providers, which accounted for half of our chat services, often lacked the resources to meet these requirements comfortably. However, we could not test this with the data collected. Further studies are needed to examine this relationship. Collaboration among service providers, knowledge exchange, and additional research are essential to move from ideal practices to practical, implementable solutions, particularly for NGOs with limited resources.

### Limitations

This study provides valuable insights into the characteristics of OCCSs and their service providers across 4 European countries, while also identifying existing gaps. However, several limitations should be acknowledged. The study was limited to 4 European countries (Belgium, Finland, Hungary, and Spain), which may have led to the omission of certain types of services present in other regions. Nevertheless, the selected countries represent different parts of Europe and encompass all major welfare system types within the European Union. Additionally, while the web search aimed to replicate a typical user’s behavior, Google’s algorithms were not controlled, meaning that implicit personalization may have introduced bias. Another limitation is that data collection relied exclusively on publicly available information from web sources. As a result, certain quality standard criteria established by Child Helpline International could not be assessed, such as the existence of standard operating procedures to guide staff or whether service providers had access to adequate funding. In cases where comparisons were possible, data were often incomplete; for example, only a small number of service providers reported the number of users served via chat, which limited the conclusions that could be drawn about service impact. Therefore, future studies should consider gathering this information through additional methods, such as surveys or interviews with professionals in the field and young service users. Moreover, due to time and resource constraints, as well as the limited prior research on this topic, the methodology focused on a selection of OCCSs. However, the representativeness of this selection could not be guaranteed. Using a stratified purposeful sampling strategy, the study included countries with varying numbers of associations and heterogeneous services, alongside countries with fewer associations that exhibited similar profiles. This approach resulted in differing saturation points. Furthermore, the Google search in Belgium was conducted solely in Dutch, which is only one of the country’s 3 official languages. Consequently, some significant chat service providers may have been overlooked. Despite these limitations, this study presents a replicable methodology that can be used to expand and deepen the existing knowledge in this area.

### Conclusions

This study provides the first comprehensive overview of the main characteristics of helpline providers and their OCCSs operating across a range of European countries. Our findings suggest that OCCSs for young people should improve transparency by sharing data on users’ contacts and address existing gaps in user privacy and accessibility, particularly for vulnerable and minority groups. Exploring existing resources and identifying potentially unmet needs are essential steps toward facilitating information exchange. Ultimately, these efforts can help address ethical and technical considerations and support the development of consensus-based practices for delivering high-quality services.

## Abbreviations

CH@T-YOUTH: Crisis Help and Assistance for Youth during Challenging Times
CQS: Core Quality Standard
LGBTIQ+: Lesbian, Gay, Bisexual, Transgender, Intersex, Queer/Questioning, and others
NGO: nongovernmental organization
OCCS: online counseling chat services
